# Cytoreductive Surgery for Peritoneal Carcinomatosis from Gastric Cancer: Technical Details

**DOI:** 10.3390/jcm10225263

**Published:** 2021-11-12

**Authors:** Thomas Boerner, Pompiliu Piso

**Affiliations:** 1 Memorial Sloan Kettering Cancer Center, Department of Surgery, 1275 York Avenue, New York, NY 10065, USA; 2 Department of Surgery, Krankenhaus Barmherzige Brüder Regensburg, Prüfeninger Straße 86, 93049 Regensburg, Germany; Pompiliu.Piso@barmherzige-regensburg.de

**Keywords:** peritoneal carcinomatosis, peritoneal metastases, gastric cancer, cytoreductive surgery, surgical technique, peritonectomy

## Abstract

Due to limited systemic treatment options, peritoneal carcinomatosis of gastric origin is still associated with a dismal outcome and is claimed a terminal disease. In the past, surgery had not been considered as a potential treatment option. However, there is emerging evidence that in selected patients, locoregional treatment modalities including cytoreductive surgery of peritoneal carcinomatosis can improve survival in patients with gastric cancer. These operative procedures are complex and challenging, and a high surgical expertise of the treating physician is necessary to prevent major postoperative morbidity and mortality with a delay of further systemic therapy. This review summarizes our current knowledge and personal experience regarding the techniques of cytoreductive surgery for peritoneal metastasis of gastric origin.

## 1. General Considerations

### 1.1. General Considerations and Extent of Resection

The indication for cytoreductive surgery (CRS) and hyperthermic intraperitoneal chemotherapy (HIPEC) for peritoneal metastatic gastric cancer is limited to patients who have synchronous peritoneal metastases with a low peritoneal cancer index (PCI) score (ideally ≤6; max of 9) [1,2,3,4] and a very high probability of achieving a complete macroscopic cytoreduction. Patients with poorly cohesive carcinoma, including signet ring cell histology, treated with CRS have been shown to have poor outcomes [2] and should not be considered good surgical candidates. Patients without response or with progression of peritoneal disease despite neoadjuvant systemic ± intraperitoneal therapy should be carefully evaluated for CRS, as they may not benefit from cytoreduction. Finally, on very rare occasions, patients with oligometastatic or locally advanced disease (e.g., local invasion of the pancreas) who have stable disease or a good response to chemotherapy may be eligible for CRS and resection of a single metastasis, owing to favorable tumor biology.

### 1.2. Technical Particularities

The parietal peritonectomy can be performed with electro-evaporative surgery using a small ball tip and high-voltage cautery for a safe resection margin and coagulation of small vessels to control bleeding. A high-flow smoke evacuator is mandatory. It is also important to avoid entering the pleural space during dissection. In some cases, the use of bipolar scissors may be helpful to control the removal of infiltrating nodules with spare resection of diaphragmatic muscle. As the procedure will also include 90 min of HIPEC administration, it may be reasonable to expedite the procedure using sealing devices.

## 2. How to Explore?

**Figure 1:**
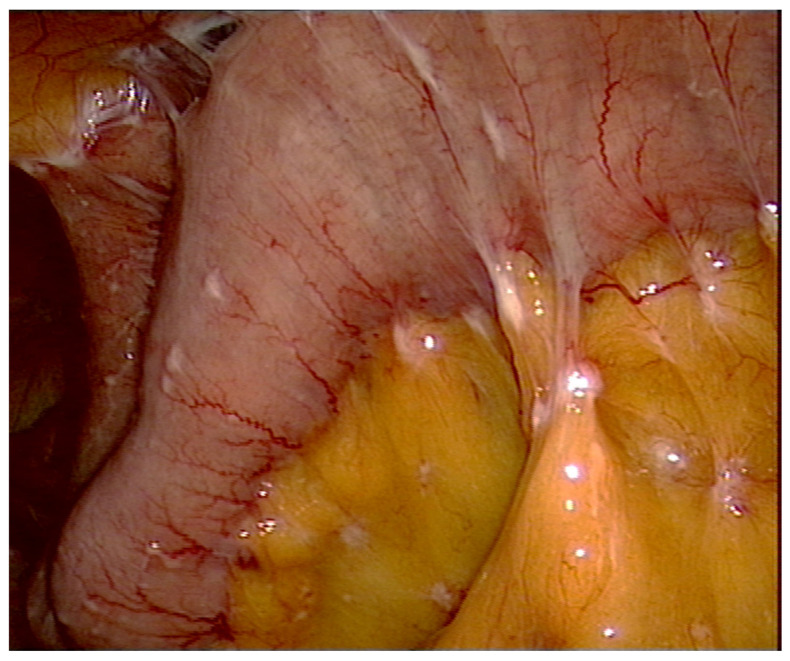
Laparoscopy demonstrating a small bowel disease with mesenteric nodules in a patient with gastric cancer. This situation cannot be treated effectively with CRS and HIPEC.

Staging laparoscopy is routinely performed prior to the CRS to assess the extent of disease and to avoid futile laparotomies (Figure 1). Even in patients with prior abdominal surgeries, a sufficient exploration of the abdominal cavity is feasible [5]. During the CRS, the abdominal cavity is entered through a midline incision from the xiphoid to the pubis. In cases of prior abdominal surgery, the old abdominal incisions are excised. We meticulously resect areas affected by the original staging laparoscopy, even when they look completely normal or consist of scar tissue, as they may contain occult viable tumor cells that could lead to future seeding metastasis. The peritoneal tumor response rate to systemic chemotherapy is low in patients with peritoneal carcinomatosis, especially in tumors of gastrointestinal origin [6,7]. In these patients, neoadjuvant chemotherapy, which is the standard treatment regimen in Europe, the United States, and many other countries [4], usually does not significantly reduce peritoneal disease. The decision to resect prior incision sites may be more difficult after neoadjuvant intraperitoneal/systemic chemotherapy (NIPS) or pressurized intraperitoneal aerosol chemotherapy (PIPAC), as these regimens have shown better effectiveness (NIPS: 62% partial response and 24% complete response rates; PIPAC: 59–90% objective clinical response rate) and result in higher conversion rates (unresectable to resectable disease) for CRS ± HIPEC [1,8,9,10,11]. In our own experience, cytoreductive procedures are more challenging to perform in patients with scarring tissue, and sclerosing areas at peritoneal sites may interfere with identifying areas affected by macroscopic tumors. Especially in patients previously treated with intraperitoneal chemotherapy, including NIPS or PIPAC, the surgical dissection may be more demanding because of adhesions or subperitoneal inflammatory reactions.

As mentioned above, tissue dissection can be performed with different instruments according to the standard of procedures of each surgical department and institution. We prefer to use bipolar scissors for most surgical procedures, but other techniques and instruments, such as dissection using ball-tip electrocautery or an ultrasonic scalpel, are also commonly used [12]. Diaphragmatic stripping can be performed more rapidly using ball-tip cautery, traction, and counter traction. If the tumor already infiltrates the superficial muscle, bipolar scissors may help to avoid a diaphragmatic resection with opening of the pleural cavity. In our own experience, the use of sealing devices can be beneficial in expediting the surgical procedure and may also lead to a significant reduction in intraoperative blood loss.

## 3. Total or Subtotal Gastrectomy?

The extent of gastric resection is determined by various factors, such as the tumor site, extent of stomach involvement, T-stage, and histology subtype, according to Lauren et al. [13]. Small tumors (T1) and those that are well differentiated can be resected with a subtotal gastrectomy, especially if they are located in the distal stomach. Generally, a macroscopic-free margin confirmed by a negative frozen section is a sufficient prerequisite for a subtotal gastrectomy. Considering the metastatic peritoneal spread in these patients and the associated poor prognosis, a subtotal resection, if locally reasonable, should be considered over a total gastrectomy due to the resultant better quality of life. However, in tumors involving the bulk of the proximal stomach or distal tumors spanning most of the lesser or greater curvature, a total gastrectomy is often inevitable to achieve negative proximal margins. Additionally, tumors with signet cell histology also often require a total gastrectomy due to the diffuse submucosal seeding that makes a complete resection via a subtotal gastrectomy very challenging [14]. As favorable outcomes are directly linked to the completeness of CRS [15], the goal in these patients is always to achieve a complete cytoreduction with the removal of all visible peritoneal disease (CC-0).

## 4. D1 or D2-Lymphadenectomy?

**Figure 2:**
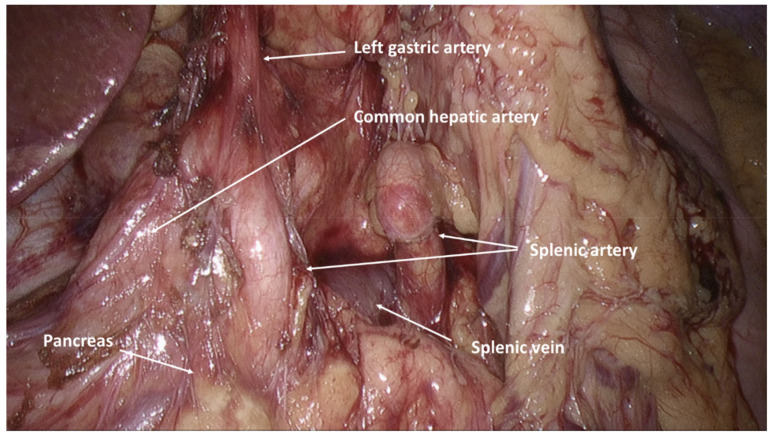
Intraabdominal view after performed pancreas- and spleen-preserving D2-lymphadenectomy.

In our opinion, if a complete cytoreduction (CC-0) can be achieved, a D2-lymphadenectomy should be the standard. There has been a longstanding and contentious debate on the optimal extent of lymphadenectomy for gastric cancer, with increasing consensus toward an extended D2 nodal dissection. D1-lymphadenectomy has been associated with less morbidity and mortality, but long-term oncologic outcomes, with better disease-specific survival, have been demonstrated in patients who undergo a D2-resection. The previous high morbidity and mortality associated with a more extended lymph node dissection were primarily attributed to the routinely performed distal pancreatectomy with concurrent splenectomy [16,17]. Due to improvements in surgical techniques, such as the introduction of the pancreas- and spleen-preserving D2-lymphadenectomy (Figure 2), morbidity and mortality have significantly decreased in recent years without compromising excellent survival outcomes [18,19,20,21].

However, CRS’s aggressive multimodal treatment approach, especially if combined with HIPEC, is still associated with substantial morbidity and mortality. Therefore, trained surgeons should only perform these large complex surgical procedures in specialized high-volume centers, as previous studies have shown that perioperative outcomes are directly related to surgical expertise and the clinical center [15,22].

Of note, the reported improved outcomes in patients treated with D2- vs. D1-lymphadenectomy, such as those reported in the Dutch D1D2 trial [23], have been exclusively generated in patients without peritoneal metastases. To our knowledge, no study has yet investigated the benefit of a more extended nodal dissection in patients with gastric cancer and peritoneal seeding. Future studies investigating the benefit of a more extended lymph node resection in patients with gastric cancer and peritoneal carcinomatosis are warranted.

## 5. How Do We Reconnect?

A Roux-en-Y esophagojejunostomy is the most preferred reconstruction approach after total gastrectomy. It is often performed with an EEA circular stapler (preferably 28 mm) and oversewn with single-stitch resorbable sutures. A loop of jejunum is mobilized to allow for a subsequent anastomosis without tension. The Roux limb should be of adequate length (40–60 cm from the downstream jejunojejunostomy) to avoid major alkaline reflux [13]. An anvil stapler is usually placed into the distal end of the esophagus and subsequently secured over the anvil by a purse-string suture. The end to side anastomosis on a Roux-Y loop is safe, as several publications have reported a very low postoperative leakage rate, even in patients who have undergone CRS with HIPEC [24]. In patients undergoing a subtotal gastrectomy, the Billroth II loop gastrojejunostomy and the Roux-en-Y gastrojejunostomy are the major reconstruction techniques of choice. The gastrojejunostomy is performed with a continuous one-layer seromuscular suture with a resorbable material. We prefer the Roux-en-Y gastrojejunostomy over the Billroth II loop anastomosis. We believe it is associated with a consistently better long-term functional outcome with lower bile reflux. The potential shortcomings of this technique, such as dumping risk or Roux stasis syndrome, have not been of major concern at our institution. This approach is supported by several prior publications, which have reported better clinical outcomes and lower postoperative morbidity in patients undergoing a Roux-en-Y gastrojejunostomy vs. a Billroth II reconstruction [25,26,27].

## 6. What Surgical Procedures to Expect?

**Figure 3:**
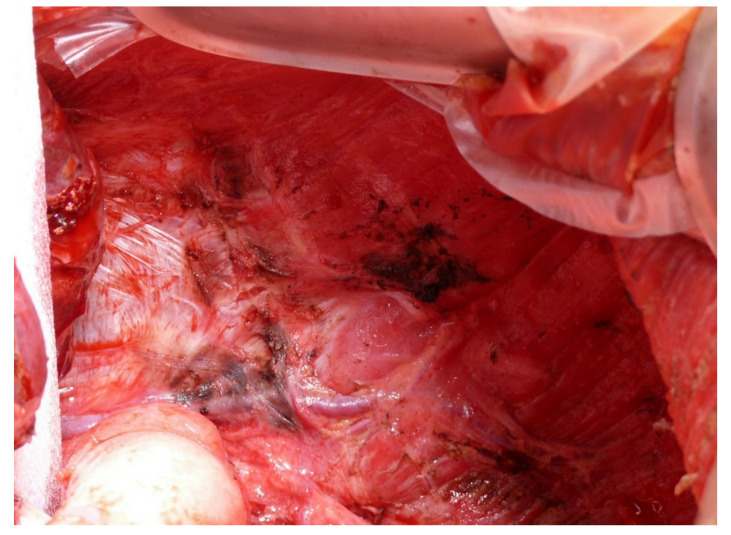
Dissected left diaphragm after parietal peritonectomy of the left upper quadrant. It is important to preserve the vessels and innervation intact, also not to enter the pleural cavity.

Indications to proceed with CRS should include good performance status and localized peritoneal disease with a PCI score ≤ 6 or a P1-stage with one quadrant affected, according to the Japanese Research Society for Gastric Cancer carcinomatosis staging (JRSGS) [15,28]. Therefore, mostly a parietal peritonectomy of the left upper quadrant with the stripping of the left diaphragm is necessary (Figure 3). A lesser omentectomy, greater omentectomy, and clearance of the hepatoduodenal ligament are additional parts of the procedure [29]. A splenectomy is seldom needed due to peritoneal metastases. Small lesions on the capsule can be destroyed by electro-evaporation using small ball-tip cautery. A concurrent bilateral salpingo-oophorectomy ± hysterectomy, as part of the routine cytoreductive effort in all women with peritoneal metastases of gastric origin, is not necessary. Bilateral oophorectomy is indicated if macroscopic or biopsy-proven microscopic disease in one of the ovaries is present; the removal of the contralateral ovary can be recommended in these cases. In our opinion, preserving unaffected ovaries in young women who have not completed their reproductive cycle should be considered. However, due to the difficulty in ascertaining microscopic involvement during surgical exploration, oophorectomies should be recommended in post-menopausal women.

## 7. Conclusions

The surgical management of patients with localized peritoneal metastases of gastric origin is very complex and challenging. These lengthy surgical procedures require extensive surgical exploration and may include the removal of multiple organs. High expertise of the treating surgeon and the medical center is crucial to ensure excellent perioperative and long-term outcomes. Therefore, treatment of these patients should be undertaken at highly specialized centers.

## Author Contributions

Conceptualization, T.B. and P.P.; methodology, T.B. and P.P.; software, T.B. and P.P.; validation, T.B. and P.P.; formal analysis, T.B. and P.P.; investigation, T.B. and P.P.; resources, T.B. and P.P.; writing—original draft preparation, T.B. and P.P.; writing—review and editing, T.B. and P.P.; visualization, T.B. and P.P.; supervision, T.B. and P.P.; project administration, T.B. and P.P.; funding acquisition, T.B. and P.P. All authors have read and agreed to the published version of the manuscript.

## Data Availability

Not applicable.
